# Sibling number and prevalence of allergic disorders in pregnant Japanese women: baseline data from the Kyushu Okinawa Maternal and Child Health Study

**DOI:** 10.1186/1471-2458-11-561

**Published:** 2011-07-14

**Authors:** Yoshihiro Miyake, Keiko Tanaka, Masashi Arakawa

**Affiliations:** 1 Department of Preventive Medicine and Public Health, Faculty of Medicine, Fukuoka University, Fukuoka, Japan; 2 Field Science for Health and Recreation, Faculty of Tourism Sciences and Industrial Management, University of the Ryukyus, Okinawa, Japan

## Abstract

**Background:**

Although an inverse relationship between number of siblings and likelihood of allergic disorders has been shown in many epidemiological studies, the biological mechanism underlying this phenomenon has not yet been identified. There is no epidemiological research regarding the sibling effect on allergic disorders in Japanese adults. The current cross-sectional study examined the relationship between number of siblings and prevalence of allergic disorders among adult women in Japan.

**Methods:**

Subjects were 1745 pregnant women. This study was based on questionnaire data. The definitions of wheeze and asthma were based on criteria from the European Community Respiratory Health Survey whereas those of eczema and rhinoconjunctivitis were based on criteria from the International Study of Asthma and Allergies in Childhood. Adjustment was made for age, region of residence, pack-years of smoking, secondhand smoke exposure at home and at work, family history of asthma, atopic eczema, and allergic rhinitis, household income, and education.

**Results:**

The prevalence values of wheeze, asthma, eczema, and rhinoconjunctivitis in the past 12 months were 10.4%, 5.5%, 13.0%, and 25.9%, respectively. A significant inverse exposure-response relationship was observed between the number of older siblings and rhinoconjunctivitis, but not wheeze, asthma, or eczema (*P *for trend = 0.03); however, the adjusted odds ratio (OR) for having 2 or more older siblings was not significant although the adjusted OR for having 1 older sibling was statistically significant (adjusted OR = 0.71 [95% CI: 0.56-0.91]). Number of total siblings and number of younger siblings were not related to wheeze, asthma, eczema, or rhinoconjunctivitis.

**Conclusions:**

This study found a significant inverse relationship between the number of older siblings and the prevalence of rhinoconjunctivitis among pregnant Japanese women. Our findings are likely to support the intrauterine programming hypothesis; however, we could not rule out the hygiene hypothesis.

## Background

Although an inverse relationship between number of siblings and likelihood of allergic disorders has been shown in many epidemiological studies, the biological mechanism underlying this phenomenon has not yet been identified [1]. Strachan first proposed the hygiene hypothesis in 1989, suggesting that exposure to infections transmitted by unhygienic contact with older or younger siblings might influence the skewing of the Th1/Th2 balance away from allergy-promoting Th2 cells toward Th1 cells, leading to a reduced risk of allergic disorders [2,3]. However, a study of young adults in the UK showed that both the number of gastrointestinal infections before the age of 5 years and the number of brothers were independently inversely related to the prevalence of atopy after mutual adjustment for the 2 factors [4].

The intrauterine programming hypothesis has been proposed as an alternative explanation of the mechanism of the sibling effect [1]. According to this theory, maternal immunomodulation acquired through multiple pregnancies might be transmitted to the developing fetus [5-7]. Although a cohort study in the USA found no association between birth order and total cord blood IgE level [8], another recent study reported a significant interaction between birth order and *IL13 *polymorphisms affecting allergic sensitization: the effect of *IL13 *was restricted only to firstborn children, suggesting adverse prenatal programming in firstborn offspring [9].

To our knowledge, there is no evidence that number of siblings affects the prevalence of allergic disorders among Japanese adults, though such evidence does exist for Japanese schoolchildren, as shown in our previous studies [10,11]. The current cross-sectional study examined the relationship between number of siblings and the prevalence of allergic disorders in Japanese pregnant women using baseline data from the Kyushu Okinawa Maternal and Child Health Study (KOMCHS).

## Methods

### Study Population

The KOMCHS is an ongoing prospective prebirth cohort study that investigates risk and preventive factors for maternal and child health problems such as allergic disorders. The KOMCHS requested that 131 obstetric hospitals in Fukuoka Prefecture, the largest prefecture on Kyushu Island in southern Japan, with a total population of approximately 5.04 million, provide as many pregnant women as possible with a set of leaflets explaining the KOMCHS, an application form to participate in the study, and a self-addressed and stamped return envelope during the period from April 2007 to March 2008. From May 2007 to March 2008, the KOMCHS also requested that 40 obstetric hospitals in Okinawa Prefecture, an island in the southernmost area of Japan, with a total population of almost 1.37 million, provide pregnant women with the same documents. In addition, to increase the sample size, from August 2007 to March 2008, pregnant women living in 6 prefectures on Kyushu Island other than Fukuoka Prefecture, with a total population of approximately 8.22 million, were provided with these documents at 252 obstetric hospitals. Pregnant women who intended to participate in the KOMCHS returned the application form to the data management center. In the end, a total of 1757 pregnant women between the 5th and 39th week of pregnancy gave their fully informed consent in writing to participate and completed the baseline survey. Excluded were 12 pregnant women because of missing data on the factors under study, leaving data on 1745 pregnant women available for analysis. The ethics committee of the Faculty of Medicine, Fukuoka University approved the KOMCHS.

### Measurements

In the baseline survey, each participant filled out a set of 2 self-administered questionnaires. Participants mailed the answered questionnaires to the data management center. Research technicians completed missing or illogical data by telephone interview.

One of the self-administered questionnaires included questions on wheeze and asthma based on the European Community Respiratory Health Survey [12] and questions on eczema and rhinoconjunctivitis based on the International Study of Asthma and Allergies in Childhood [13,14]. Wheeze was defined as a positive response to the question, 'Have you had wheezing or whistling in your chest at any time in the last 12 months?' Asthma was defined as either having experienced an asthma attack during the last 12 months or currently using asthma medication. Affirmative answers to the following 3 questions were required to indicate the presence of eczema: 'Have you ever had an itchy rash which was coming and going for at least 6 months?', 'Have you had this itchy rash at any time in the last 12 months?' and 'Has this itchy rash at any time affected any of the following places: the folds of the elbows, behind the knees, in front of the ankles, under the buttocks, or around the neck, ears, or eyes?' Rhinoconjunctivitis was defined as a positive response to the following 2 questions: 'In the last 12 months, have you had a problem with sneezing or a runny or blocked nose when you did not have a cold or flu?' and 'In the last 12 months, has this nose problem been accompanied by itchy-watery eyes?' The questionnaire also elicited information on age, number of older and younger siblings, smoking habits, secondhand smoke exposure at home and at work, family history of asthma, atopic eczema, and allergic rhinitis, household income, and education. A family history of asthma, atopic eczema, or allergic rhinitis (including Japanese cedar pollinosis) was considered to be present if one or more parents or siblings of the study subjects had been diagnosed by a physician with any of these allergic disorders.

The second questionnaire was a validated self-administered diet history questionnaire. Data regarding diet were not used in the present study.

### Statistical analysis

Age, region of residence, pack-years of smoking, secondhand smoke exposure at home and at work, family history of asthma, atopic eczema, and allergic rhinitis, household income, and education were *a priori *selected as potential confounding factors. Our previous study using baseline data from the Osaka Maternal and Child Health Study showed that current smoking was significantly associated with a higher prevalence of asthma whereas secondhand smoke exposure was positively significantly related to the prevalence of allergic rhinitis [15]. Region of residence was classified into 3 categories (Fukuoka Prefecture, other than Fukuoka Prefecture on Kyushu Island, and Okinawa Prefecture), pack-years of smoking into 3 (none, 0.05-3.9, and ≥ 4.0), secondhand smoke exposure at home and at work into 2 (never and ever), household income into 3 (< 4,000,000, 4,000,000-5,999,999, and ≥ 6,000,000 yen/year), education into 3 (< 13, 13-14, and ≥ 15 years), total number of siblings into 4 (0, 1, 2, and ≥ 3), number of older siblings into 3 (0, 1, and ≥ 2) and number of younger siblings into 3 (0, 1, and ≥ 2). Age was used as a continuous variable.

Multiple logistic regression analysis was performed to estimate adjusted odds ratios (ORs) and 95% confidence intervals (CIs) of allergic disorders relative to the number of siblings. Trend of association was assessed by a logistic regression model assigning consecutive integers to the categories of the exposure variables. All statistical analyses were performed using the SAS software package version 9.2 (SAS Institute, Inc., Cary, NC, USA).

## Results

The prevalence values of wheeze, asthma, eczema, and rhinoconjunctivitis in the past 12 months were 10.4%, 5.5%, 13.0%, and 25.9%, respectively, among the 1745 pregnant women. The mean age of the participants at baseline was 31.2 years. Family history of allergic rhinitis was much more common among participants than were family history of asthma or family history of atopic eczema (Table 1). About 10% had 3 or more siblings.

**Table 1:** Distribution of selected characteristics in 1745 pregnant women, Kyushu Okinawa Maternal and Child Health Study, Japan

Variable	n	%
Region of residence		
Fukuoka Prefecture	970	55.6
Other than Fukuoka Prefecture in Kyushu	593	34.0
Okinawa Prefecture	182	10.4
Pack-years of smoking		
None	1182	67.7
0.05-3.9	263	15.1
≥ 4.0	300	17.2
Ever secondhand smoke exposure at home	1315	75.4
Ever secondhand smoke exposure at work	1105	63.3
Family history of asthma	330	18.9
Family history of atopic eczema	302	17.3
Family history of allergic rhinitis	759	43.5
Household income (yen/year)		
< 4,000,000	632	36.2
4,000,000-5,999,999	620	35.5
≥ 6,000,000	493	28.3
Education (years)		
< 13	429	24.6
13-14	576	33.0
≥ 15	740	42.4
Total no. siblings		
0	94	5.4
1	788	45.2
2	688	39.4
≥ 3	175	10.3
No. older siblings		
0	829	47.5
1	622	35.6
≥ 2	294	16.9
No. younger siblings		
0	695	39.8
1	695	39.8
≥ 2	355	20.3

Table 2 presents adjusted ORs and their 95% CIs for allergic disorders in relation to the number of siblings. Larger total sibship size was not materially related to the prevalence of wheeze, asthma, eczema, or rhinoconjunctivitis. A significant inverse exposure-response relationship was observed between number of older siblings and the prevalence of rhinoconjunctivitis (*P *for trend = 0.03); however, the adjusted OR for having 2 or more older siblings was not statistically significant, although the adjusted OR for having 1 older sibling was statistically significant (adjusted OR = 0.71 [95% CI: 0.56-0.91]). No independent associations were found between the number of older siblings and the prevalence of wheeze, asthma, or eczema. There were no evident relationships between the number of younger siblings and any of the outcomes under investigation.

**Table 2:** Adjusted odds ratios (ORs) and 95% confidence intervals (CIs) for allergic disorders according to the number of siblings in 1745 pregnant women, Kyushu Okinawa Maternal and Child Health Study, Japan^1^

	Wheeze		Asthma		Eczema		Rhinoconjunctivitis	
				
	Prevalence	OR (95% CI)	Prevalence	OR (95% CI)	Prevalence	OR (95% CI)	Prevalence	OR (95% CI)
Total siblings								
0 (n = 94)	9.6%	1.00	5.3%	1.00	11.7%	1.00	26.6%	1.00
1 (n = 788)	11.0%	1.07 (0.53-2.40)	5.3%	0.94 (0.39-2.82)	11.8%	0.84 (0.45-1.74)	27.0%	0.87 (0.54-1.46)
2 (n = 688)	9.9%	0.88 (0.43-1.99)	5.7%	0.92 (0.37-2.78)	13.4%	0.94 (0.49-1.94)	25.3%	0.79 (0.48-1.32)
≥ 3 (n = 175)	10.3%	0.75 (0.32-1.87)	5.7%	0.72 (0.24-2.45)	17.1%	1.31 (0.63-2.92)	22.9%	0.75 (0.42-1.38)
*P *for trend		0.18		0.55		0.18		0.23
Older siblings								
0 (n = 829)	10.7%	1.00	5.1%	1.00	12.7%	1.00	29.0%	1.00
1 (n = 622)	11.1%	1.02 (0.72-1.44)	6.6%	1.22 (0.77-1.93)	12.5%	1.03 (0.74-1.41)	22.8%	0.71 (0.56-0.91)
≥ 2 (n = 294)	8.2%	0.74 (0.45-1.20)	4.4%	0.79 (0.40-1.49)	14.6%	1.33 (0.89-1.98)	23.8%	0.77 (0.56-1.06)
*P *for trend		0.34		0.77		0.21		0.03
Younger siblings								
0 (n = 695)	9.8%	1.00	5.9%	1.00	12.8%	1.00	23.6%	1.00
1 (n = 695)	11.1%	1.07 (0.75-1.53)	4.5%	0.72 (0.44-1.17)	11.7%	0.78 (0.56-1.09)	27.8%	1.22 (0.95-1.56)
≥ 2 (n = 355)	10.4%	0.90 (0.57-1.39)	6.8%	1.02 (0.59-1.75)	15.8%	1.09 (0.74-1.58)	26.8%	1.18 (0.87-1.59)
*P *for trend		0.71		0.86		0.91		0.21

^1 ^Adjustment for age, region of residence, pack-years of smoking, secondhand smoke exposure at home and at work, family history of asthma, atopic eczema, and allergic rhinitis, household income, and education.

When we stratified our subjects into two groups based on the presence or absence of older siblings, subjects with 1 or more older siblings had a significantly lower prevalence of rhinoconjunctivitis than subjects without older siblings did, but there was no such association between the presence of older siblings and wheeze, asthma, or eczema: the adjusted OR was 0.73 (95% CI: 0.59-0.91). Neither the presence of younger siblings nor the presence of siblings of any age was related to any of the outcomes under study.

## Discussion

The current study found a significant inverse exposure-response relationship between the number of older siblings and the prevalence of rhinoconjunctivitis, but not wheeze, asthma, or eczema. Total sibship size and number of younger siblings were not materially associated with the prevalence of wheeze, asthma, eczema, or rhinoconjunctivitis.

Epidemiological evidence for the sibling effect on allergic disorders among adults is limited. A cross-sectional study of men and women in the UK aged 16-30 years found significant inverse exposure-response associations of sibship size and birth order with the prevalence of hay fever whereas larger sibship size, but not birth order, was significantly inversely related to the prevalence of eczema-urticaria [16]. In a cross-sectional study of pregnant women in Denmark, larger sibship size was significantly associated with a lower prevalence of allergic rhinitis and asthma with allergic rhinitis, but not asthma without allergic rhinitis; the inverse associations between having older siblings and allergic rhinitis and asthma with allergic rhinitis were more pronounced than the inverse associations between having younger siblings and these disorders [17]. Having 4 or more older siblings was significantly related to a lower prevalence of allergic rhinitis and/or allergic conjunctivitis, but not asthma or atopic dermatitis, over the lifetime of the subjects in a cross-sectional study of Finnish young adults aged 18-24 years [18]. In a retrospective cohort study of Swedish male conscripts, number of older siblings was significantly inversely associated with the prevalence of allergic rhinitis, but not asthma [19].

Turning to the evidence concerning Japanese children, having 2 or more older siblings was significantly related to a lower prevalence of rhinoconjunctivitis, but not wheeze or eczema, in a cross-sectional study of children aged 12-15 years [10]. Another cross-sectional study of Japanese children aged 6-15 years reported significant exposure-response relationships between larger sibship size and the prevalences of wheeze, asthma, eczema, and rhinoconjunctivitis, and demonstrated that having older siblings reduces the prevalences of eczema and rhinoconjunctivitis while having younger siblings reduces only the prevalence of eczema [11]. These findings, which demonstrate an inverse association between the number of older siblings and allergic rhinitis and null associations between sibship size and wheeze, asthma, or eczema, are in agreement with the current results.

Karmaus and colleagues have found that the level of cord blood IgE was reduced with increasing birth order and that elevated cord blood IgE values were significantly related to an increased risk of allergic sensitization at 48 months of age, though there was no association between birth order and allergic sensitization [5]. Karmaus and colleagues have also shown that maternal IgE levels are significantly reduced with increasing numbers of live offspring [6]. In a study in the UK, women with more pregnancies over a seven-year period were more likely to have lost atopy and symptoms of hay fever, but not asthma, during that period [7]. The fact that maternal immune tolerance against allergens increases as more live offspring are born, and the fact that maternal immune status is communicated to the fetus, might explain the inverse association between the number of older siblings and rhinoconjunctivitis in the current study. Another version of the intrauterine programming hypothesis suggests a potential role of hormones; progesterone and testosterone seem to promote the preferential development of Th2-like cells [1]. In addition, the potential role of endocrine disruptors should be discussed. Parity was significantly associated with decreasing levels of organochlorines in breast milk of Norwegian women [20]. Higher levels of cord blood dichlorodiphenyldichloroethylene were significantly related to an increased risk of asthma at 6.5 years in a cohort study in Spain [21]. Nevertheless, a UK study cited above showed a significant inverse association between sibship size and the prevalence of combined allergies, asthma, eczema-urticaria, and hay fever among people born between 1918 and 1930 when organochlorines had not come into widespread use [16].

The absence of a sibling effect on wheeze, asthma, or eczema in this study might be ascribed to the attenuation of the sibling effect with advancing age. In our previous study, larger sibship size was significantly associated with the prevalences of wheeze, asthma, eczema, and rhinoconjunctivitis among children aged 6-10 years, but only with the prevalence of eczema among subjects aged 11-15 years [11]. It should be noted that the results of that study, specifically, the findings concerning eczema and rhinoconjunctivitis in the older age group, are at variance with the present findings.

This study had certain methodological advantages. Study subjects were homogeneous in that they were all pregnant. Definitions of wheeze and asthma were based on the questions in the European Community Respiratory Health Survey. Likewise, definitions of eczema and rhinoconjunctivitis were based on the questions in the International Study of Asthma and Allergies in Childhood, although validation tests of such questions have not been performed for young Japanese adults. Several potential confounders were adjusted for, though we could not control for breastfeeding, early day care center attendance, and external factors as aeroallergens and air pollution.

This study also had certain limitations. The participation rate could not be calculated because the exact number of eligible pregnant women who were provided with the KOMCHS documents and application form by the 423 collaborating obstetric hospitals is not available. Nevertheless, the participation rate must have been fairly low, given that the present study used data from only 970 pregnant women who lived in Fukuoka Prefecture, while, according to the government of Fukuoka Prefecture, the number of childbirths was 46,393 in 2007 and 46,695 in 2008. We were not able to assess differences between participants and non-participants because information on personal characteristics such as age, socioeconomic status, and history of allergic disorders was not available for non-participants. Our subjects were probably not a representative sample of Japanese women in the general population. For example, educational levels were higher in the current study population than in the general population. According to the 2000 population census of Japan, the proportions of women aged 30 to 34 years in Fukuoka Prefecture with years of education of < 13, 13-14, ≥ 15, and unknown were 52.0%, 31.5%, 11.8%, and 4.8%, respectively [22]. The corresponding figures for the current study were 24.6%, 33.0%, 42.4%, and 0.0%, respectively. The present population therefore might have had greater awareness about health issues compared to the general population. The lack of significant inverse relationships between total sibship size and the prevalences of wheeze and eczema might be attributable to an insufficient statistical power.

The interface between allergy/immunology and pregnancy should be discussed, as it may have an influence on the association of interest. It has been suggested that pregnancy involves a shift to the Th2 side of the immune response [23] although Chaouat et al. have pointed out the importance of the role of natural killer cells and IL-12, IL-15, and IL-18 tripods in successful or failed pregnancies in humans beyond the Th1/Th2 paradigm [24]. The hormonal changes in pregnancy are often invoked to explain the apparent association between rhinitis symptoms and pregnancy. However, rhinitis ascribed solely to pregnancy may not be a distinct entity because most pregnant women do not have significant nasal symptoms [23].

## Conclusion

The current study among Japanese women found a significant inverse relationship between the number of older, but not total or younger, siblings and the prevalence of rhinoconjunctivitis, whereas no such sibling effect was observed for wheeze, asthma, and eczema. Our findings are likely to support the intrauterine programming hypothesis, though we could not rule out the hygiene hypothesis.

## Competing interests

The authors declare that they have no competing interests.

## Authors' contributions

All authors contributed to the study concept and design and the acquisition of data. YM was responsible for the analysis and interpretation of data and the drafting of the manuscript. All authors participated in critically revising the manuscript and approved the final version of the manuscript.

## Pre-publication history

The pre-publication history for this paper can be accessed here:

http://www.biomedcentral.com/1471-2458/11/561/prepub
